# Sequence Determinants for Nuclear Retention and Cytoplasmic Export of mRNAs and lncRNAs

**DOI:** 10.3389/fgene.2018.00440

**Published:** 2018-10-17

**Authors:** Alexander F. Palazzo, Eliza S. Lee

**Affiliations:** Department of Biochemistry, University of Toronto, Toronto, ON, Canada

**Keywords:** TREX, lncRNAs, transposable elements, RNA modification, splicing, polyadenylation, constructive neutral evolution

## Abstract

Eukaryotes are divided into two major compartments: the nucleus where RNA is synthesized and processed, and the cytoplasm, where mRNA is translated into proteins. Although many different RNAs are made, only a subset is allowed access to the cytoplasm, primarily RNAs involved in protein synthesis (mRNA, tRNA, and rRNA). In contrast, nuclear retained transcripts are mostly long non-coding RNAs (lncRNAs) whose role in cell physiology has been a source of much investigation in the past few years. In addition, it is likely that many non-functional RNAs, which arise by spurious transcription and misprocessing of functional RNAs, are also retained in the nucleus and degraded. In this review, the main sequence features that dictate whether any particular mRNA or lncRNA is a substrate for retention in the nucleus, or export to the cytoplasm, are discussed. Although nuclear export is promoted by RNA-splicing due to the fact that the spliceosome can help recruit export factors to the mature RNA, nuclear export does not require splicing. Indeed, most stable unspliced transcripts are well exported and associate with these same export factors in a splicing-independent manner. In contrast, nuclear retention is promoted by specialized *cis*-elements found in certain RNAs. This new understanding of the determinants of nuclear retention and cytoplasmic export provides a deeper understanding of how information flow is regulated in eukaryotic cells. Ultimately these processes promote the evolution of complexity in eukaryotes by shaping the genomic content through constructive neutral evolution.

## Introduction

The distinguishing feature of eukaryotic cells is that they are divided into two compartments: the nucleus where pre-messenger RNAs (mRNAs) are made and processed, and the cytoplasm where mature mRNAs are translated into proteins (Martin and Koonin, 2006; Palazzo and Gregory, 2014). This is in contrast to prokaryotes, where mRNAs are made and translated at the same time in the same compartment. In eukaryotes, the temporal and spatial separation of mRNA synthesis from translation allows each newly made RNA to be subjected to extensive quality control before it ever encounters a ribosome (Palazzo and Akef, 2012). This quality control involves the nuclear retention and/or degradation of spurious transcripts, which are synthesized from intergenic DNA regions, and misprocessed RNAs, which result from errors in splicing or 3′ cleavage. In the absence of this quality control, spurious transcripts and misprocessed mRNAs would be exported to the cytoplasm and then translated into toxic proteins. Thus, the separation of RNA synthesis in the nucleus and translation in the cytoplasm, and the associated quality control mechanisms that go along with this separation, reduces some of the harmful side-effects of non-functional RNAs that are transcribed from non-functional DNA. This is why both junk DNA and a low level of spurious transcription are tolerated in most eukaryotes (Palazzo and Gregory, 2014; Palazzo and Lee, 2015).

Importantly, non-functional RNAs, whose harmful effects are reduced by eukaryotic quality control systems, are not effectively eliminated by natural selection and some of these can eventually evolve into functional long non-coding RNAs (lncRNAs). These add to the repertoire of bio-active polymers that organisms can use to regulate growth, homeostasis and development. Although some lncRNAs function in the cytoplasm, most operate in the nucleus (Djebali et al., 2012; Derrien et al., 2012; Tilgner et al., 2012; Palazzo and Gregory, 2014; Kaewsapsak et al., 2017). As a result, lncRNAs must be appropriately sorted to allow for their proper retention in the nucleus or export to the cytoplasm. This separation is critical as alterations in mRNA nuclear retention and cytoplasmic export have been associated with various diseases (Borden and Culkovic-Kraljacic, 2018; Bovaird et al., 2018). Furthermore, many neuropathological states are associated with the formation of RNA-protein liquid–liquid phase separated structures that can disrupt proper nuclear-cytoplasmic trafficking by soaking up nuclear transport factors and components of the nuclear pore complex (Mahboubi et al., 2013; Zhang et al., 2018).

**FIGURE 1:**
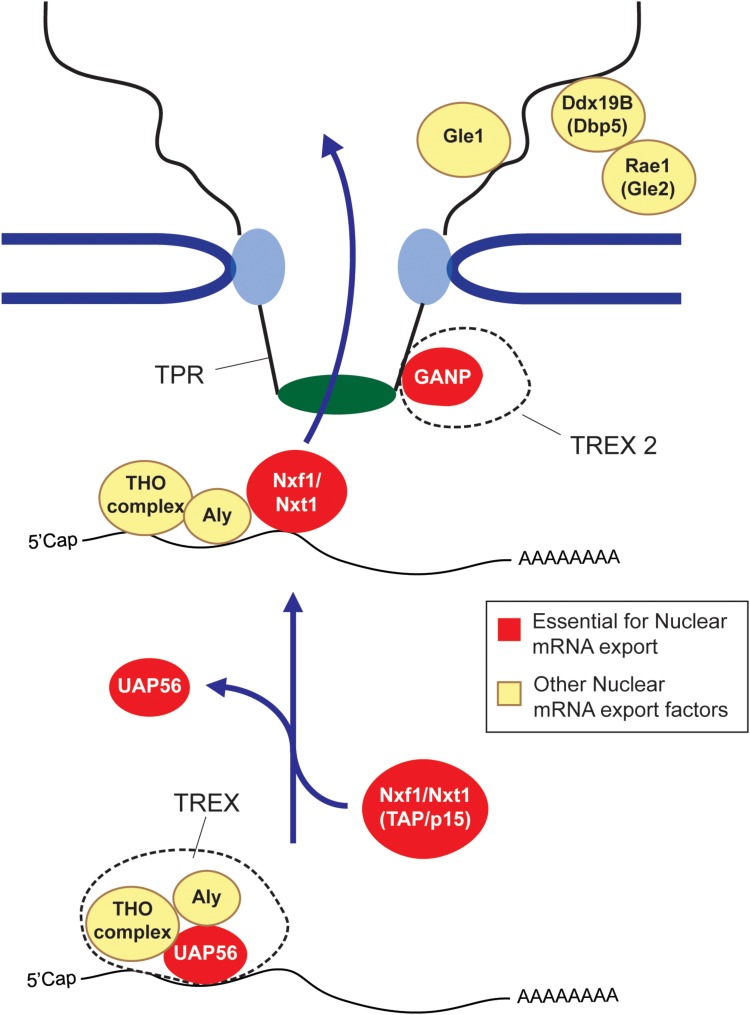
The mRNA nuclear export pathway. The TREX complex, which is composed of the Tho complex, the RNA helicase UAP56 (or its paralog URH49) and Aly are loaded onto the mRNA co-transcriptionally or by processing events. At some point, the UAP56 hydrolyses ATP and then is replaced by the nuclear export receptor composed of Nxf1 and Nxt1 (also known as TAP and p15) to form an export competent mRNP. The Nxf1-Nxt1 heterodimer physically interacts with the FG repeats of Nups to ferry its cargo across the nuclear pore. GANP, which forms part o the TREX2 complex is also required for export, although its exact role is not understood. After passing through the nuclear pore complex, the mRNP is furthered remodeled by cytoplasmic pore-associated proteins such as Gle1, Dbp5 and Rae1/Gle2. It is though that these remodeling events remove certain nuclear associated exported factors, which are then recycled back into the nuclear pore. In some cases these mRNP remodeling events render the mRNP more ‘translationally’ competent (Palazzo and Truong, 2016). Factors that are essential for mRNA export are depicted in red, other export factors are depicted in yellow.

So how does this all work? First, long and short RNAs are generally treated very differently. In mammals, RNA length appears to be evaluated by hnRNP C (McCloskey et al., 2012), with transcripts that are shorter than 200 nucleotides (e.g., snRNAs, tRNAs, and miRNAs) being directed toward specialized export pathways (Masuyama et al., 2004; Fuke and Ohno, 2008; McCloskey et al., 2012), while longer RNAs (mRNA and lncRNAs) being shunted to a more generalized pathway that require the major export complex, TREX, and its heterodimeric nuclear transport receptor composed of Nxf1/TAP and Nxt1/p15 (**Figure 1**) (Katahira et al., 1999; Strässer et al., 2002). In addition to these major export factors, other export-promoting complexes exist. SR proteins, which promote splicing, also help to recruit Nxf1/TAP to these RNAs (Huang et al., 2003; Müller-McNicoll et al., 2016). TREX2, which is thought to localize to the nucleoplasmic side of the nuclear pore, also plays a major role in promoting export (Wickramasinghe et al., 2010, 2014; Umlauf et al., 2013; Zhang et al., 2014b). Dbp5, Rae1/Gle1, and Gle2, which associate with the cytoplasmic face of the nuclear pore, may be involved in recycling nuclear export factors back into the nucleoplasm (Blevins et al., 2003; Lund and Guthrie, 2005; Alcázar-Román et al., 2006; Weirich et al., 2006), although this is not quite understood. For reviews on these factors and complexes see (Katahira, 2012; Palazzo and Akef, 2012; Heath et al., 2016; Borden and Culkovic-Kraljacic, 2018). The mechanism that dictates nuclear retention is less well understood. Some of the factors involved are described in later sections of this review.

So how are exported RNAs, which mostly code for protein, differentiated from nuclear retained RNAs, which are typically non-coding? Ultimately these two types of RNAs must differ in one or more ways. This can include *cis*-elements (i.e., particular RNA motifs) or general features such as splicing, polyadenylation, and RNA modifications. These differences will dictate what proteins are loaded onto the RNA, resulting in the formation of a ribonucleoprotein (RNP) complex that determines the ultimate fate of the transcript.

In this review we shall cover what is known about the RNA features that impact the nuclear retention and cytoplasmic export of mRNAs and lncRNAs. However, before we start, there are a few points to keep in mind. First, although we speak of a given RNA species as being retained in the nucleus or exported to the cytoplasm, few RNAs are completely nuclear or cytoplasmic at steady state. Instead, each RNA species exists at some point along a spectrum between these two extremes. Second, the ultimate steady state distribution of an RNA is dictated not only by the rate of RNA export, but also by the rates of RNA synthesis and of RNA decay in both the nucleus and the cytoplasm. Few studies have taken all these various factors into account with some exceptions. Having said that, it is clear that nuclear retention and cytoplasmic export play critical roles in dictating the ultimate distribution of any RNA species. Third, although it is generally true that most mRNAs are well exported, many are not (Djebali et al., 2012; Bahar Halpern et al., 2015; Bouvrette et al., 2018). Likewise, although there is a general consensus that many lncRNAs are nuclear, it is also clear that several are cytoplasmic, with some studies suggesting that the number of cytoplasmic lncRNAs may be higher than previously thought (Wilk et al., 2016; Bouvrette et al., 2018).

## What Is the Default Pathway?

Before addressing the question of what sequence determinants impact nuclear export, it becomes necessary to determine whether an RNA which lacks any distinguishing feature is a substrate for nuclear export. In other words, what is the default pathway – nuclear retention or cytoplasmic export? Three pieces of evidence point to the fact that long RNAs do not require any specialized *cis*-element for them to be exported from the nuclei of mammalian tissue culture cells.

### Reporter mRNAs

Whether the default pathway for any given long RNA was nuclear retention or cytoplasmic export was up for debate for a number of years, due largely to differences between the nuclear/cytoplasmic distribution of mRNAs derived from different reporter genes (Luo and Reed, 1999; Lu and Cullen, 2003; Masuyama et al., 2004; Nott et al., 2004; Palazzo et al., 2007; Valencia et al., 2008; Lei et al., 2011, 2013; Takemura et al., 2011; McCloskey et al., 2012). For example, it had been observed that certain reporter mRNAs transcribed from cDNAs were not exported, suggesting that in the absence of splicing, mRNAs are nuclear retained (Valencia et al., 2008). This confusion was largely due to the fact that it was unclear whether any particular reporter is truly devoid of *cis*-elements or other distinguishing features that may promote or inhibit mRNA nuclear export. More recently, we have demonstrated that two widely used reporters, a mini gene derived from the Drosophila *fushi tarazu* gene (*ftz*), and the *β-globin* mRNA, each have nuclear retention elements (Akef et al., 2015; Lee et al., 2015). Importantly, when the newly identified nuclear retention elements were removed, RNAs generated from these reporters were well exported despite the fact that they are not spliced.

### mRNAs With Random Sequences

In other experiments it was found that RNAs generated from artificial genes, purported to have “random” sequences, were not exported but were instead rapidly degraded (Dias et al., 2010). One potential problem with completely random sequences is that they contain elevated numbers of CG dinucleotides, which are depleted in vertebrate genomes (Karlin and Mrázek, 1997). In DNA, CG dinucleotides are often methylated, and when these N^5^-methylcytosines undergo spontaneous deamination they are converted to thymidine causing CG dinucleotides to be mutated away in vertebrates (Lindahl, 1993). In contrast, unmethylated cytosines deaminate to uracils, which are efficiently removed by uracil-DNA glycosylase and reconverted back to cytosines. Recently it was found that RNAs with significant numbers of CG dinucleotide are substrates for decay, which would effectively prevent their accumulation in the cytoplasm (Takata et al., 2017). This process likely evolved to protect cells against viral infection. Interestingly, the proteins involved in this decay, ZAP/ZC3HAV1 and TRIM25, are primarily cytoplasmic and are known to be involved in viral RNA degradation.

In the study by Dias et al. (2010), the RNA reporters with “random” sequence were in fact generated from the reverse compliment sequences of intronless genes from the human genome (*IFNA1, IFNB1* and *HSPB3*). As expected, the three constructs have relatively low CG-content (as is true for almost all human-derived DNA); however, all three are predicted to have either 5′ splice site motifs or 3′ splice site motifs [scoring ≥ 0.97 according to NNSPLICE 0.9 (Reese et al., 1997)]. These motifs are known to inhibit nuclear export if they are not used for splicing (see The 5′ Splice Site Motif – Other Intron-Associated Motifs). It is also possible that these transcripts were spliced and that the researchers were detecting the distribution of lariat introns in their experiments. Additionally, it is conceivable that these RNAs may have other nuclear retention elements. Again, interpreting experiments with “random” RNAs is difficult, as unidentified *cis*-elements may drastically alter the behaviors of these transcripts.

### lncRNAs

The last piece of evidence which suggests that nuclear export is the default pathway is that when nuclear localized lncRNAs were analyzed, it was observed that they contained nuclear retention elements (Miyagawa et al., 2012; Zhang et al., 2014a; Lubelsky and Ulitsky, 2018; Shukla et al., 2018). When these nuclear retention elements were removed or mutated, the altered lncRNAs were exported. In one extreme case the intronless *MALAT1* lncRNA was expressed as a series of small fragments (each ∼1 kb), with the majority of the resulting RNAs being efficiently exported (Miyagawa et al., 2012). This allowed researchers to identify two regions that retain this lncRNA in the nucleus by targeting it to nuclear speckles. Moreover, fusion of either of these two nuclear retention fragments to reporters promotes their nuclear retention (Lubelsky and Ulitsky, 2018; Shukla et al., 2018). Thus, it is likely that lncRNAs like *MALAT1* must be actively retained in the nucleus, and in the absence of these factors, the resulting RNAs are automatically exported to the cytoplasm.

Taking in all of these lines of evidence, it is likely that in the absence of any active *cis*-element, a stable RNA that is capped and polyadenylated is a substrate for nuclear export.

## The Role of RNA Processing in Nuclear Retention and mRNA Export

In eukaryotes, most functional RNAs are extensively processed. Although very strong processing signals are found in regions of the genome that are used to produce functional RNA transcripts (be they mRNAs or lncRNAs), weaker processing signals are found throughout the genome. Even comparing mRNAs and lncRNAs, the former are typically more efficiently spliced than the latter (Tilgner et al., 2012; Melé et al., 2017; Mukherjee et al., 2017; Deveson et al., 2018). Thus, robust processing is typically a good indication that the RNA transcript in question is functional and likely encoding a protein (Palazzo and Akef, 2012; Palazzo and Lee, 2015). Moreover, many RNA processing machineries directly interact with, and promote the recruitment of, RNA nuclear export factors. This “coupling” between RNA processing and RNA nuclear export has been extensively documented in other reviews (Maniatis and Reed, 2002; Moore and Proudfoot, 2009; Palazzo and Akef, 2012).

### Splicing

Splicing involves the removal of introns by the spliceosome, which in turn can deposit factors onto the newly spliced RNA. By comparing the localization of these spliced RNAs to transcripts synthesized from cDNAs (which lack introns), it has been observed that splicing in some scenarios enhances the extent and the rate of nuclear export (Luo and Reed, 1999; Palazzo et al., 2007; Valencia et al., 2008). The spliceosome directly interacts with many key mRNA nuclear export factors, such as the TREX component UAP56 (Fleckner et al., 1997; Strässer and Hurt, 2001). Indeed, splicing is known to help recruit TREX components to RNAs (Masuda et al., 2005; Dufu et al., 2010; Chi et al., 2013). This phenomenon is, however, not universal. Most cDNA-derived RNAs (which lack introns) are well exported (Palazzo and Akef, 2012) and can recruit TREX and Nxf1/TAP (Taniguchi and Ohno, 2008; Hautbergue et al., 2009; Akef et al., 2013, 2015; Lee et al., 2015). Likely, where splicing matters most is in transcripts that happen to have nuclear retention elements. In some cases, splicing can override their activity (Akef et al., 2015), while in other cases it cannot (Lee et al., 2015). The second scenario is probably true for lncRNAs that are efficiently spliced and yet still retained in the nucleus (Hacisuleyman et al., 2014).

### 5′ Capping

The 5′ RNA cap is an N^7^-methylguanine connected via a 5′ to 5′ triphosphate linkage to the beginning of RNAs which are generated by RNA Polymerase II. This structure recruits the nuclear cap binding complex (CBC), which consists of CBP20 and CBP80. It has been reported that CBP80 can recruit nuclear export factors, such as the TREX component Aly, to the 5′ end of spliced (Cheng et al., 2006) and intronless (Nojima et al., 2007) transcripts. More recently it was shown that a functional paralog of CBP80, NCBP3, also interacts with components of the TREX and exon junction complexes (Gebhardt et al., 2015). Importantly, the co-depletion of CBP80 and NCBP3 inhibits mRNA nuclear export (Gebhardt et al., 2015). As such it is clear that the 5′ cap is a major contributor to the proper export of mRNAs. Whether it is absolutely required is a bit unclear. The incorporation of non-canonical caps (trimethyl-guanosine [3mGpppG], adenosine [ApppG]) does not block the export of certain microinjected intronless RNAs, but does block the export of intron-containing mRNAs (Palazzo et al., 2007). Since the 5′cap is also required for splicing (Izaurralde et al., 1994), it is possible that RNAs with cap analogs are inefficiently spliced and are thus actively retained in the nucleus. As detailed below, RNA motifs that are associated with introns are potent nuclear retention signals. Lastly, it has been reported that the export of circular RNAs requires UAP56 (Huang et al., 2018), a core factor of the TREX complex that is required for the export of most mRNAs (Luo et al., 2001; Strässer and Hurt, 2001; Kapadia et al., 2006). This would suggest that TREX-mediated export does not strictly require a 5′ cap to function.

### 3′ Cleavage and Polyadenylation

The 3′ end of an RNA Polymerase II-generated transcript is recognized and processed by the cleavage and polyadenylation complex (Chan et al., 2011). Members of this complex interact with Aly (Johnson et al., 2009; Shi et al., 2017), the TREX component THOC5 (Katahira et al., 2013; Tran et al., 2014) and Nxf1/TAP (Ruepp et al., 2009). In line with these studies, the RNAs produced from reporter genes with defective 3′ cleavage signals are restricted to the nucleus (Dias et al., 2010). It is, however, likely that these RNAs are never released from RNA polymerase due to the lack of cleavage, complicating the interpretation of this observation. In another set of experiments, it was found that microinjected RNAs that lack a poly(A)-tail, but are modified at their 3′end to protect the RNAs from degradation are retained in the nucleus (Akef et al., 2013). Again, it is possible that the modification itself, a dialdehyde formed by the oxidation of the free 3′ end ribose by periodate, may trigger nuclear retention. On the flip side, a GFP reporter that lacks a tail and contains a 3′ terminal triple helix structure derived from the *MALAT1* lncRNA, which stabilizes unpolyadenylated transcripts, is efficiently exported (Wilusz et al., 2012). This observation suggests that either the poly(A)-tail is not strictly required for mRNA export or that this triple helix motif promotes nuclear export, although this element is derived from *MALAT1*, a nuclear lncRNA. Another observation that suggests that the poly(A)-tail is not absolutely required for export is that circular RNAs, which lack a tail, are efficiently exported in a UAP56-dependent manner (Huang et al., 2018). Finally, histone mRNAs, which do not have a poly(A)-tail, are exported by Nxf1 and do not appear to have any export-promoting *cis*-elements (Erkmann et al., 2005).

In summary, it is likely that RNA processing helps to promote export; however, results from a variety of case studies (cDNA derived reporters, GFP mRNA with a 3′ terminal triple helix, and circular RNAs) suggest that these processes are not absolutely required. Again, as most RNAs exist on a spectrum between being fully nuclear and being fully cytoplasmic, RNA processing events may help to move the RNA closer to the cytoplasmic end of this continuum.

## The Role of RNA Nucleotide Modifications in Nuclear Retention and mRNA Export

It has been known for quite some time that RNA is extensively modified; however, until recently the majority of these studies focused on these modifications within tRNA and rRNA. More recently it has been observed that mRNA and lncRNAs are also modified. Furthermore, some of these modifications appear to impact nuclear export.

### Adenosine to Inosine Editing

Adenosine to inosine editing was the first RNA modification known to affect nuclear export. Specifically, it was observed that double stranded RNA (dsRNA) was a substrate for the RNA specific adenosine deaminase (ADAR), which converts adenosine to inosine (Polson et al., 1991). This reaction occurs specifically in the nucleus and promotes the nuclear retention of these RNAs (Zhang and Carmichael, 2001). Thus RNAs that are prone to forming long dsRNA, including mRNAs with inverted Alu repeats and viruses (Kumar and Carmichael, 1997; Athanasiadis et al., 2004; Blow et al., 2004; Kim et al., 2004; Levanon et al., 2004), are modified and retained in paraspeckles (Chen et al., 2008). In certain cases nuclear retention of inosine-containing mRNAs can also be used to regulate gene expression (Prasanth et al., 2005). Interestingly, this nuclear retention pathway appears to be less active in human embryonic stem cells due to the fact that they do not express the lncRNA *NEAT1*, which is required for paraspeckle formation (Chen and Carmichael, 2009).

### Other RNA Modifications

In the last 6 years, it has become clear that other modifications, which were known to occur in tRNA and rRNA, play significant roles in mRNA biology. This includes N^6^-methyladenosine (Dominissini et al., 2012; Meyer et al., 2012), which accumulates near the stop codon, N^5^-methylcytosine (Squires et al., 2012) and N^1^-methyladenosine (Dominissini et al., 2016; Li et al., 2016a), which both accumulate near the start codon, and pseudouridine (Carlile et al., 2014; Schwartz et al., 2014; Li et al., 2015), which accumulates in the ORF and 3′UTR.

Recently, it has been reported that N^6^-methyladenosine promotes the nuclear export of mRNAs (Roundtree et al., 2017) through the action of the YTHDC1 protein, which directly binds to the modified base and helps to recruit nuclear export factors to the mRNA. This makes sense as depletion of the N^6^-methyladenosine demethylase, ALKBH5, enhances overall mRNA export (Zheng et al., 2013). Similarly, N^5^-methylcytosine has also been reported to promote mRNA nuclear export by recruiting Aly to the transcript (Yang et al., 2017). Although N^1^-methyladenosine has not been directly linked to export, this modification is enriched in the 5′ terminal exon of a particular class of mRNAs (Cenik et al., 2017). These mRNAs have interesting 5′ terminal exons. Not only are they modified, but they also tend to contain the start codon (in most human genes the start codon is found in internal exons), and are enriched in certain GC-rich motifs that are associated with exon junction complexes (Singh et al., 2012). Typically, exon junction complexes are deposited upstream of all newly formed exon-exon splice sites; however, in a subset of genes the exon junction complex also associates with these GC-rich motifs. Importantly, this complex has also been found to bind to nuclear export factors (Le Hir et al., 2001; Singh et al., 2012), although it is not strictly required for export (Palazzo et al., 2007).

In conclusion, RNA modifications that have been reported to promote export may enhance this process, especially if an RNA has nuclear retention elements; however, it is likely that RNA modifications are not absolutely required to promote export.

## The Role of *cis*-Elements in Nuclear Retention and mRNA Export

### The 5′ Splice Site Motif

**FIGURE 2:**
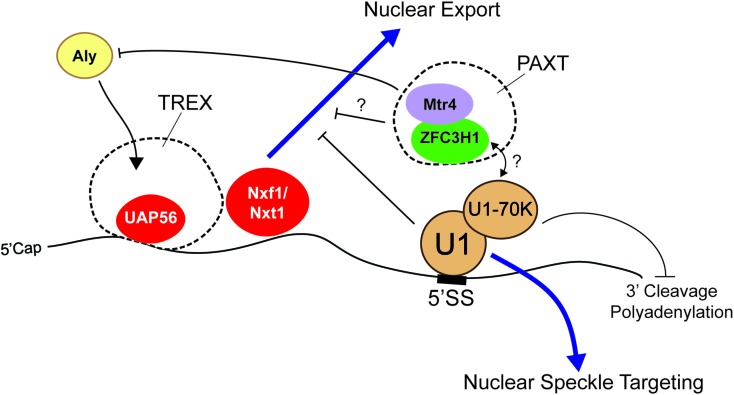
The 5′ splice site motif promotes nuclear retention of RNAs. The presence of the 5′ splice site (5′SS) motif in the 3′ untranslated region (3′UTR) promotes nuclear retention. U1 snRNP recognizes the motif and may recruit nuclear surveillance machinery (e.g., PAXT complex) through U1-70K. Interestingly, although these RNAs are able to recruit UAP56 and Nxf1/Nxt1, they are not exported. Mtr4, a member of the PAXT complex and a co-activator/co-adaptor of the nuclear exosome, competes with Aly for its association with the RNA and various 5′ cap binding proteins. Other work suggests that the 5′ splice site motif suppresses premature cleavage and polyadenylation through the action of U1-70K.

Some of the most studied RNA motifs that affect the distribution and stability of mature mRNA are the 5′ and 3′ splice site motifs, which specify the boundaries of introns. These are typically removed by the act of splicing. Importantly, these motifs are found in fully processed exported RNAs of many viruses, such as HIV. In its normal life cycle, HIV produces both spliced and unspliced RNAs from the same primary transcript, the latter being used to make late-stage proteins and to generate the RNA-based genome that will be incorporated into new viruses that are assembled in the cytoplasm of the host cell. Importantly, these unspliced RNAs are retained in the nucleus in early stages by the presence of intronic sequences (Chang and Sharp, 1989; Lu et al., 1990; Borg et al., 1997; Séguin et al., 1998). These retention signals can be overcome in late stages by the virally encoded Rev protein, which recognizes the Rev response element, an RNA structure that is present in the late stage RNAs and the viral RNA genome (Chang and Sharp, 1989; Emerman et al., 1989; Tan et al., 1996). In the absence of Rev, the nuclear retention of these RNAs was mediated in part by U1 snRNP, the component of the spliceosome that recognizes the 5′ splice site motif (Lu et al., 1990) (**Figure 2**). It should be noted that the Rev response element itself also contributes to the nuclear retention of the late-stage viral mRNAs and of the HIV genomic RNA when Rev protein is not present (Brighty and Rosenberg, 1994; Nasioulas et al., 1994).

In other work, it was also demonstrated that when the 5′ splice site motif was present in the terminal exon of an mRNA, it inhibited expression of the encoded protein. This was due in part to the fact that this element suppresses 3′ polyadenylation, which in turn targets the mRNA for degradation (Gunderson et al., 1998) (**Figure 2**). This configuration is not only seen in certain viral mRNAs, but also in human mRNAs. For example, a mutation in the LAMTOR2 gene, which is associated with congenital neutropenia, creates a novel 5′ splice site in the 3′ UTR that results in the inhibition of gene expression (Langemeier et al., 2012). Importantly, this inhibition is likely due to the recruitment of U1 snRNP to the mature mRNA (Langemeier et al., 2012), through the direct hybridization of the U1 snRNA with the 5′ splice site. Indeed, when the sequence of the U1 snRNA is altered so that it now base pairs to some other mRNA, these newly targeted transcripts becomes silenced (Fortes et al., 2003; Abad et al., 2008; Goraczniak et al., 2009; Blázquez and Fortes, 2013). A protein component of the U1 snRNP, U1-70K, is required for this inhibition by directly interacting and inhibiting poly(A)-polymerase (Gunderson et al., 1998).

As we stated in the introduction, disentangling the effects of mRNA stability and nuclear retention on the final nuclear/cytoplasmic distribution of an mRNA can be challenging. This is certainly the case with the 5′ splice site motif which appears to promote both RNA degradation and RNA nuclear retention. To tease these two forces apart, we monitored the level and distribution of newly synthesized reporter mRNAs that contained or lacked a 5′ splice site motif in its 3′UTR. This was accomplished by microinjecting DNAs that were transcribed into each mRNA species, then allowing transcription to proceed for a short amount of time (15–20 min) before halting transcription with α-amanitin, and then monitoring the newly transcribed RNA by fluorescence *in situ* hybridization at various timepoints after injection. Using this approach we found that about half of newly synthesized reporter mRNAs that contained a 5′ splice site motif are rapidly degraded, with the remaining fraction being retained in the nucleus as polyadenylated RNAs (Lee et al., 2015) (**Figure 2**). Interestingly, the nuclear retained RNAs accumulate in nuclear speckles, subnuclear regions where post-transcriptional splicing is thought to occur (Dias et al., 2010; Vargas et al., 2011). Indeed, unspliced mRNAs which are generated by the inhibition of the U2 or U4 snRNPs, also accumulate in nuclear speckles (Kaida et al., 2007; Hett and West, 2014). These unspliced RNAs and 5′ splice site bearing RNAs are likely targeted to nuclear speckles by U1. Then, the subsequent failure to complete the splicing reaction prevents these RNAs from exiting the nuclear speckles. In agreement with these results, the artificial tethering of U1-70K to a reporter RNA prevents its nuclear export, although the authors did not test for nuclear speckle targeting (Takemura et al., 2011). Surprisingly, 5′ splice site motif-containing mRNAs are still able to recruit UAP56 and Nxf1/TAP (Lee et al., 2015), suggesting that if they could reach the pore, these mRNAs could cross it; however, access to the pore may be prevented by their sequestration into speckles (**Figure 2**). This may explain why many poorly exported mRNAs are also localized to nuclear speckles (Bahar Halpern et al., 2015).

The presence of 5′ splice site motifs may also be critical for the nuclear retention of many lncRNAs and may help to distinguish them from mRNAs. According to annotated databases of human genes, fully mature lncRNAs, unlike mature mRNAs, are not depleted of 5′ splice site motifs in their terminal exons (Lee et al., 2015). Even when comparing intronless RNAs, lncRNAs have higher levels of 5′ splice site motifs than mRNAs (Lee et al., 2015). These numbers may be an underestimate as lncRNAsare not as efficiently spliced as mRNAs (Tilgner et al., 2012; Melé et al., 2017; Mukherjee et al., 2017; Deveson et al., 2018), with many isoforms containing retained introns due to the inefficient recruitment of spliceosomal factors to 3′ splice sites (Melé et al., 2017). Interestingly, the corresponding 5′ splice sites of these inefficiently spliced introns still recruit U1 (Melé et al., 2017), and thus likely promote nuclear retention. Indeed, lncRNA splicing appears to be sloppier than mRNA splicing, with each lncRNA gene producing a multitude of different isoforms with altered splice junctions (Deveson et al., 2018), and this may also cause the inclusion of 5′ splice site motifs into the mature RNA.

**FIGURE 3:**
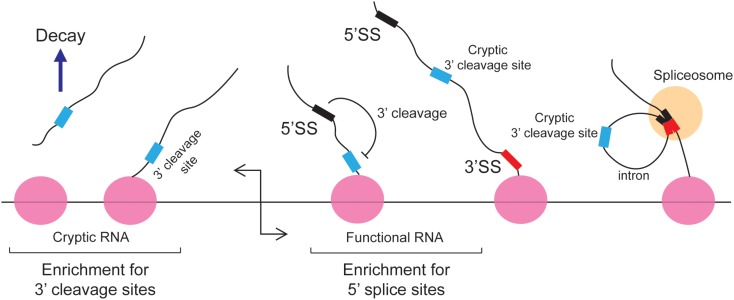
The 5′ splice site motif suppresses premature cleavage and polyadenylation. Almada et al. (2013) found that in bidirectional promoters that produce one stable transcript, 5′SS motif are enriched in the sense direction (stable RNA) while 3′ cleavage sites are enriched in the anti-sense direction (unstable RNA). Under normal circumstances these cryptic unstable RNAs are cleaved and degraded. In the sense direction, the 5′ splice site motif suppresses the use of downstream cryptic 3′ cleavage sites, allowing RNA PolII to synthesize the RNA transcript without the recruitment of the 3′ end processing machinery. These cryptic 3′ cleavage sites are typically present in introns and are removed during splicing.

The 5′ splice site motif also inhibits 3′ cleavage. When cells were depleted of U1 snRNPs, prematurely truncated mRNAs started to appear (Kaida et al., 2010; Berg et al., 2012; Almada et al., 2013). This was due to a decrease of splicing which led to the appearance of retained introns, which in turn contained cryptic 3′ cleavage/polyadenylation sites that were inappropriately used by the 3′ cleavage machinery. Importantly, these truncated RNAs contain intact 5′ splice site upstream of the new 3′ end. It was inferred that under normal circumstances the binding of U1 inhibits 3′ cleavage from any sites in the downstream intron (**Figure 3**). This finding is in agreement with studies of Bovine Papilloma Virus and HIV RNAs where the recruitment of U1 to a 5′ splice site inhibited proximal 3′ cleavage events (Furth et al., 1994; Ashe et al., 1995, 1997; Vagner et al., 2000). Similar results were seen with the mutant form of the LAMTOR2 mRNA (Langemeier et al., 2012). It should be noted that in normal situations, suppression of 3′ cleavage by U1 snRNP helps to perform two tasks: first it represses the misprocessing of mRNAs by preventing the activity of cryptic 3′ cleavage/polyadenylation sites that are found in introns; secondly, it enforces promoter directionality. In particular, it was found that in bidirectional promoters which generate an unstable short cryptic transcript in one direction and a stable protein-coding mRNA in the other direction, that 3′ cleavage/polyadenylation consensus sites were enriched in the former, and 5′ splice site motifs were enriched in the latter (Almada et al., 2013) (**Figure 3**). Under normal conditions the transcriptional elongation of these cryptic transcripts is curtailed by the presence of these 3′ cleavage/polyadenylation sites. Early 3′ cleavage promotes RNA degradation, although the exact mechanism in vertebrates remains unclear (Proudfoot, 2016). In contrast, the 5′ splice site motif present on the opposite transcriptional unit prevents the utilization of any downstream cryptic 3′ cleavage/polyadenylation site and thus promotes the transcriptional extension and stability of functional RNAs. This arrangement of 5′ splice site motifs and 3′ cleavage/polyadenylation sites is sometimes referred to as the U1-PAS axis (PAS stands for polyadenylation sites).

One important complex which may promote nuclear retention and degradation of RNAs that contain 5′ splice site motifs is the PAXT complex, which consists of the RNA helicase, Mtr4, the zinc finger-containing protein, ZFC3H1, and the nuclear poly(A) binding protein, PABPN1 (Meola et al., 2016) (**Figure 2**). Depletion of Mtr4 or ZFC3H1 resulted in the cytoplasmic accumulation of truncated mRNAs that utilized cryptic 3′ cleavage sites from intronic regions (Ogami et al., 2017). Mtr4 may promote nuclear retention of these transcripts by competing with the RNA export adaptor Aly for binding of the 5′ cap (Fan et al., 2017). Furthermore, Mtr4 is also a co-activator of the nuclear exosome (Schilders et al., 2007), the major RNA degradation machinery in the nucleus, suggesting that PAXT may also target these RNAs for degradation. It is currently unclear how the PAXT complex would recognize its substrates, although one possibility is that it interacts with U1 that is bound to misprocessed mRNAs.

In budding yeast, mRNAs with unspliced introns are also nuclear retained and degraded, and this likely requires an intact 5′ splice site (Legrain and Rosbash, 1989). This retention activity requires the Mlp1/2 proteins (Galy et al., 2004; Vinciguerra et al., 2005), which form the nuclear basket, a structure that sits on the nucleoplasmic face of the nuclear pore complex. In vertebrates, the nuclear basket protein TPR, which shares some homology with Mlp1/2, is also required for the nuclear retention of intron-bearing mRNAs (Coyle et al., 2011; Rajanala and Nandicoori, 2012). Interestingly, TPR is also required for mRNA export (Shibata et al., 2002; Umlauf et al., 2013; Wickramasinghe et al., 2014), likely by associating with the TREX2 factor GANP (**Figure 1**), which is essential for the nuclear export of most mRNAs (Wickramasinghe et al., 2010; Zhang et al., 2014b).

Finally, it should be pointed out that the nuclear retention of mRNAs harboring retained introns may also be used to regulate gene expression. It has been found that certain regulated mRNAs contain “detained” introns that are poorly spliced, leading to the retention of the transcripts into the nucleus (Boutz et al., 2015; Mauger et al., 2016; Naro et al., 2017). These are typically the last introns in the pre-mRNA, and it is likely that the primary signal for nuclear retention is the presence of a 5′ splice site motif in these terminal exons. These retained mRNAs are stable and not subject to degradation. However, in response to some signal, these introns are post-transcriptionally spliced, releasing the mRNAs from the nucleus and triggering protein production.

### Other Intron-Associated Motifs

Besides the 5′ splice site motif, it has been reported that other sequences that are normally associated with introns also potentiate nuclear retention. Typically, the 3′ end of an intron is defined by a polypyrimidine track which can be recognized by the polypyrimidine track binding protein (PTB). Association of PTB with mature RNAs is known to inhibit splicing and nuclear export (Yap et al., 2012; Roy et al., 2013). In addition, the 3′ end of the intron also recruits the splicing factor U2AF65, whose association with a mature RNA also promotes nuclear retention (Takemura et al., 2011). Finally, it also appears that the presence of an intact branch-point sequence in the mature mRNA also promotes nuclear retention in budding yeast (Legrain and Rosbash, 1989; Rain and Legrain, 1997). Thus, it is likely that several different intron-associated elements may help to promote the nuclear retention and decay of RNA.

### Transposable Element Associated Motifs

The majority of the human genome is composed of dead transposable elements, constituting half to two-thirds of all DNA (Gregory, 2005; de Koning et al., 2011). Although they are numerous, they are rarely found in mature mRNAs and found at moderate levels in lncRNAs (Kelley and Rinn, 2012). When they are present, they usually inhibit nuclear export and promote RNA decay. As described above, if a pair of transposable elements are found in the sense and anti-sense orientation in a single transcript, they can hybridize to form double stranded RNAs. These regions either become substrates for the ADAR enzyme and thus acquire inosine modifications (Chen et al., 2008; Chen and Carmichael, 2008), or are recognized by the RNA binding protein Staufen, which targets these RNAs for decay (Gong and Maquat, 2011; Elbarbary et al., 2013; Park and Maquat, 2013; Lucas et al., 2018). In addition, double stranded RNAs activate the kinase PKR, which then phosphorylates the translation initiation factor eIF2α and thus shuts down global translation (Clemens and Elia, 1997). Typically, PKR is activated by double stranded viruses, however, it is also known to regulate the processing of certain host mRNAs (Ilan et al., 2017). It remains unclear if PKR activity impacts nuclear export.

It is likely that other features associated with transposable elements are recognized by nuclear retention machinery. It was recently found that the reverse complement of the Alu SINE, a primate-specific transposable element, contains a 42 nucleotide long element, named SIRLOIN, that mediates nuclear retention by recruiting the RNA binding protein, hnRNP K (Lubelsky and Ulitsky, 2018). A similar C-rich motif that contributed to nuclear retention was found in a large analysis of human lncRNAs (Shukla et al., 2018). Since Alu elements are not found outside of primates, lncRNAs must use other elements, especially in non-primates. In addition, it appears that many transposable elements are recognized by particular C2H2 zinc finger proteins (Emerson and Thomas, 2009; Rowe and Trono, 2011; Schmitges et al., 2016), many of which contain not only the capability to bind DNA, but also RNA (Burdach et al., 2012). It has been speculated that when a new transposable element invades a genome, it catalyzes the evolution of novel zinc finger proteins that protects the host. These zinc finger proteins likely repress transposable element activity primarily through transcriptional silencing, although it is also possible that these proteins may help target RNAs for decay or nuclear retention.

### Other *cis*-Elements That Promote Nuclear Retention

A few other *cis*-elements that promote nuclear retention have been characterized in the literature. As mentioned above, the Rev responsive element promotes nuclear retention (Brighty and Rosenberg, 1994; Nasioulas et al., 1994). Another example is the AGCCC motif which promotes the nuclear retention of the *BORG* lncRNA (Zhang et al., 2014a). Although the authors of this study show that the presence of this motif correlated with the nuclear/cytoplasmic distribution of a few lncRNAs and mRNAs, such a sequence would be predicted to be depleted from mRNAs in general; however, in a large genome-wide analysis, we have failed to detect such a depletion (A. F. Palazzo, unpublished observations). This is unlike the 5′ splice site motif, which is depleted from intronless mRNAs and the 3′ terminal exons of human mRNAs (Lee et al., 2015).

In many cases, recruitment of certain proteins to the RNA has been linked to nuclear retention, however, it remains unclear whether the simple presence of their RNA-binding motifs promotes retention more broadly throughout the transcriptome. This is true of the *Firre* lncRNA, whose nuclear retention requires the recruitment of hnRNP U protein (Hacisuleyman et al., 2014). Similarly, it has been reported that the recruitment of hnRNP A2 inhibits nuclear export (Lévesque et al., 2006). Again, a more global analysis of how these factors affect the nuclear/cytoplasmic distribution of all RNAs would be useful in determining whether other nuclear retention elements exist.

Other global analyses have tried to identify nuclear retention/export motifs by sequencing RNA derived from nuclear and cytoplasmic compartments (Bahar Halpern et al., 2015; Bouvrette et al., 2018). Interestingly, both studies found a reasonable number of mRNAs that were poorly exported. Although the distribution of mRNAs with either the nuclear or cytosolic compartment correlated with the association of certain RNA binding proteins, no obvious patterns were discovered. This is in contrast to lncRNAs where the presence of motifs that are either associated with transposable elements (Lubelsky and Ulitsky, 2018; Shukla et al., 2018) or unused splicing signals (Lee et al., 2015; Melé et al., 2017) likely promote widespread nuclear retention. Why would lncRNAs and mRNAs have different mechanisms for their nuclear distribution? One difference may be that nuclear lncRNAs are actively retained while nuclear mRNAs are simply exported to the cytoplasm at a very low rate. This would allow these particular mRNAs to accumulate in the nucleus at high levels. It has been hypothesized that since these large pools of nuclear mRNAs would slowly exit the nucleus, they would supply the cytoplasm with a steady level of mRNA over long periods of time and this could help to buffer the protein translation machinery in the cytoplasm from any wide fluctuations in mRNA production in the nucleus (Bahar Halpern et al., 2015). This may be especially important for genes that experience transcriptional bursts, the sporadic production of many mRNAs in a short interval, followed by periods of inactivity (Larson, 2011). Without this buffering, mRNA levels in the cytoplasm would stochastically increase and decrease over short intervals of time, especially if the mRNA has a short half-life.

A few studies have uncovered large RNA elements that have nuclear retention activity but remain ill-defined. Two of the best examples are the intronless *β-globin* mRNA and the *MALAT1* lncRNA. In the case of *β-globin*, the nuclear retention activity maps to the last 210 nucleotides of the open reading frame (Akef et al., 2015). This nuclear retention activity can be overcome by either extending the length of the transcript (Akef et al., 2015), including an intron to promote splicing (Valencia et al., 2008; Akef et al., 2015), or by inserting certain export-promoting viral RNA elements (Guang and Mertz, 2005; Chi et al., 2014). Deleting the first or the second half of this 210 nucleotide region does not disrupt nuclear retention, suggesting that there may be multiple sequences that account for this activity. Despite this, the two halves do not share any obvious motif or structure. In the case of *MALAT1*, its two nuclear speckle targeting regions (termed regions “E” and “M”) are also ill-defined (Miyagawa et al., 2012). In the case of region E which is about 1KB in length, elimination of the first or last half disrupts its activity. For region M, its activity maps to 600 nucleotides, but it is disrupted if it is truncated any further. It is likely that the *XIST, NEAT1* and *TUG1* lncRNAs also have large nuclear retention elements (Lubelsky and Ulitsky, 2018; Shukla et al., 2018). Ultimately, it remains possible that these pieces of RNA contain one or more discrete motifs or structures that have weak nuclear retention activity (Shukla et al., 2018), and that further in-depth studies would be needed in order to better define these elements.

### *Cis*-Elements That Promote Nuclear Export

Many viral elements are known to promote nuclear export; however, a number of these act to overcome nuclear retention elements such as the presence of unspliced introns. Besides the Rev responsive element (described above), the most well studied is the constitutive transport element (CTE) of type D retroviruses (Bray et al., 1994). This large-structured RNA directly recruits Nxf1/TAP to the transcript (Grüter et al., 1998). Interestingly, the *Nxf1* mRNA contains a CTE-like element that can also recruit Nxf1/TAP (Li et al., 2006; Wang et al., 2015). These elements appear to modulate the export of *Nxf1* mRNA isoforms that contain a retained intron (and hence a 5′ splice site). The mRNA is then translated into a short isoform of Nxf1 that may play a role in mRNA trafficking (Li et al., 2016b).

Some mRNAs have been described to have *cis*-elements that promote nuclear export. mRNAs that encode proteins required for the cell cycle, contain an export promoting element in their 3′UTR which consists of a stem loop structure that recruits the eIF4E protein (Culjkovic et al., 2005, 2006). Intriguingly, the export of these transcripts requires UAP56, but not Nxf1/TAP (Topisirovic et al., 2009). Instead they use the CRM1 nuclear transport receptor, which promotes the export of proteins. The recruitment of HuR to mRNAs and lncRNAs has also been reported to promote their nuclear export (Fan and Steitz, 1998; Noh et al., 2016). Finally, it has been reported that naturally intronless transcripts contain specialized cytoplasmic accumulation region elements (CAR-E), which recruit specific complexes to the RNA (Lei et al., 2011, 2013). Some of the interpretations of these experiments are complicated by the fact that CAR-Es were fused to reporters harboring nuclear retention elements whose activity can be overcome by simply extending the length of the transcript (see Discussion in Akef et al., 2015). Notably, the export of these mRNAs require the TREX component UAP56, which appears to be recruited to reporter RNAs that do not contain any known nuclear export elements (Taniguchi and Ohno, 2008; Akef et al., 2015; Lee et al., 2015). Thus, the functional relevance of these purported export-promoting elements seems unclear at this time. It is likely that *bone fide* export-promoting elements, such as the CTE, function by overcoming the activity of nuclear retention elements, such as the ones present in mRNAs with retained introns.

## Nuclear Retention And Export Of RNAs, A Force For Constructive Neutral Evolution?

### The Conversion of Junk RNA to lncRNA

The nuclear/cytoplasmic distribution of RNAs plays an important role in shaping the genomic content of eukaryotes by evolution. In particular, the nuclear retention and degradation of spurious transcripts eliminates much of the harm caused by junk RNA and hence reduces the deleteriousness of cryptic TSSs and intergenic DNA regions that harbor such sites (Palazzo and Akef, 2012; Palazzo and Gregory, 2014; Palazzo and Lee, 2015). As a result, junk DNA and its associated junk RNA are not effectively eliminated by natural selection. It is likely that these non-functional transcripts act as the raw substrates for natural selection and some are converted into novel functional lncRNAs. Thus, in a sense, junk RNA and functional lncRNAs come as a package. The idea that neutral mutations (i.e., intergenic insertions, and the serendipitous creation of cryptic TSSs) create novel entities (i.e., junk RNA) that are subsequently shaped by natural selection to create novel genes (i.e., lncRNAs) is an example of a general process called *constructive neutral evolution* (Stoltzfus, 1999, 2012; Gray et al., 2010; Lukeš et al., 2011). A key component in this process is the role of the nuclear/cytoplasmic division (and its associated quality control mechanisms) in reducing the deleteriousness of spurious transcription.

**FIGURE 4:**
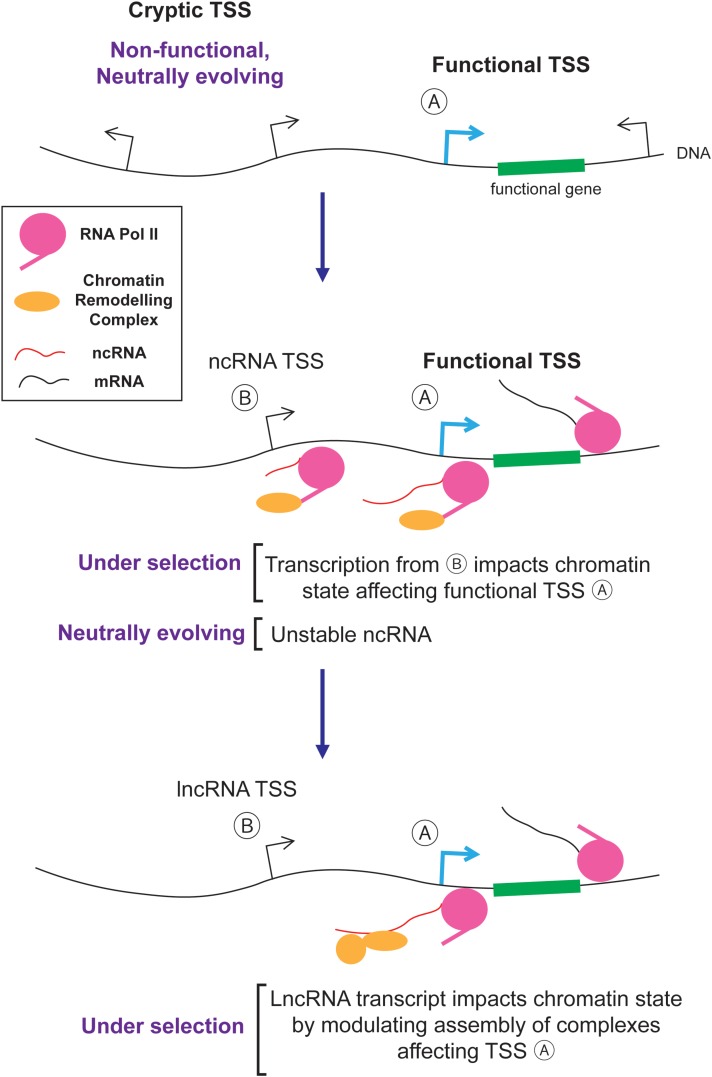
The evolution of lncRNAs from junk RNAs.

So how exactly would junk RNA be converted to lncRNA? Likely, this is a step by step process where new entities are created by non-adaptive processes and then acquire functions which can be selected for by natural selection. One example is presented in **Figure 4**. First, random mutations create and destroy cryptic TSSs. These sites are engaged by RNA polymerase II which not only generates unstable ncRNAs, but also recruits histone modification enzymes that alter chromatin packaging downstream from the TSS (van Werven et al., 2012; Castelnuovo et al., 2013; Kim et al., 2016; Woo et al., 2017). If the resulting altered histone modifications impart some benefit by regulating nearby genes in a way that improves the fitness of the organism, then the transcriptional event and its cryptic TSS will be selectively retained. Eventually the ncRNA generated from these loci, which is initially a by-product, may act as a platform to help assemble chromatin remodeling complexes in the vicinity of their target genes. In this way, the ncRNA acquires a novel function over time and is thus converted into a lncRNA (**Figure 4**).

This conversion process may frequently occur in tissues that have a high amount of spurious transcription, such as in developing spermatids (Kaessmann, 2010; Jandura and Krause, 2017). During sperm development, DNA is unpackaged from histones and then repackaged into protamines. This transiently exposed DNA can act as a non-specific substrate for RNA polymerases causing high levels of spurious transcription. Once a ncRNAs acquires some associated function in the testes, it can subsequently be expressed in other tissues. This is known as the “out of the testes” hypothesis (Kaessmann, 2010; Jandura and Krause, 2017).

### The Conversion of Misprocessing to Alternative Processing

The nuclear/cytoplasmic distribution and degradation of RNA also facilitates the evolution of alternative splicing. In particular, by retaining and degrading misprocessed mRNAs, they are not efficiently translated into proteins and do not cause much harm to the organism. This reduces the deleteriousness of splicing and polyadenylation errors and prevents their elimination by natural selection. This may explain why splicing appears to be inherently sloppy in mammalian cells. In support of this idea, it has been widely noted that although most genes are alternatively spliced, they typically give rise to only one polypeptide (Tress et al., 2017), suggesting that many spliced isoforms are not translated due to their degradation and/or nuclear retention. As such, nuclear/cytoplasmic distribution and degradation of RNA prevents the elimination of cryptic splice site motifs and any other splicing-regulating elements that may appear by random mutation in the genome. These elements then act as the raw substrates necessary for the evolution of functional alternative splicing events. This is another example of constructive neutral evolution in action. In this case the newly created entities are splice sites and/or elements that regulate splicing, which are rendered effectively neutral by the RNA nuclear retention and degradation machinery, and these provide the raw substrates for the evolution of alternatively spliced isoforms. A similar process can be invoked for the evolution of 3′ cleavage/polyadenylation sites.

## Conclusion

Results from ENCODE point to a wide diversity of nuclear/cytoplasmic distribution for many different types of RNA molecules (Djebali et al., 2012; Palazzo and Gregory, 2014). Over the past few years, we have gained a fuller picture of the rules that dictate RNA distribution to these two compartments. We have established that any stable long RNA is a substrate for nuclear export unless it contains a nuclear retention element. Undoubtably, splicing and other RNA processing events further enhance nuclear export. In addition, RNA modifications also play an important role in this process. Although our understanding of the major components that drive export are well known, we still must identify nuclear retention complexes and determine their mode of action to obtain a full picture of how the nuclear and cytoplasmic transcriptomes are achieved.

## Author Contributions

All authors listed have made a substantial, direct and intellectual contribution to the work, and approved it for publication.

## Conflict of Interest Statement

The authors declare that the research was conducted in the absence of any commercial or financial relationships that could be construed as a potential conflict of interest.
